# Polarization Independent Metamaterial Absorber with Anti-Reflection Coating Nanoarchitectonics for Visible and Infrared Window Applications

**DOI:** 10.3390/ma15103733

**Published:** 2022-05-23

**Authors:** Ahmad Musa, Mohammad Lutful Hakim, Touhidul Alam, Mohammad Tariqul Islam, Ahmed S. Alshammari, Kamarulzaman Mat, M. Salaheldeen M., Sami H. A. Almalki, Md. Shabiul Islam

**Affiliations:** 1 Pusat Sains Ankasa (ANGKASA), Institut Perubahan Iklim, Universiti Kebangsaan Malaysia, Bangi 43600, Selangor, Malaysia; p117238@siswa.ukm.edu.my (A.M.); p108762@siswa.ukm.edu.my (M.L.H.); 2 Department of CSE, International Islamic University Chittagong (IIUC), Kumira, Chattogram 4318, Bangladesh; 3 Department of Electrical, Electronic and Systems Engineering, Faculty of Engineering and Built Environment, Universiti Kebangsaan Malaysia, Bangi 43600, Selangor, Malaysia; kamarulzaman@ukm.edu.my; 4 Electrical Engineering Department, College of Engineering, University of Ha’il, Ha’il 81481, Saudi Arabia; ahm.alshammari@uoh.edu.sa; 5 Department of Electrical Engineering, Faculty of Energy Engineering, Aswan University, Aswan 81528, Egypt; mohamedsalah40@hotmail.com; 6 Department of Electrical Engineering, College of Engineering, Taif University, P.O. Box 11099, Taif 21944, Saudi Arabia; s.h.almalki@tu.edu.sa; 7 Faculty of Engineering (FOE), Multimedia University, Persiaran Multimedia, Cyberjaya 63100, Selangor, Malaysia; shabiul.islam@mmu.edu.my

**Keywords:** metamaterial absorber, anti-reflection coating, optical window, infrared window

## Abstract

The visible and infrared wavelengths are the most frequently used electromagnetic (EM) waves in the frequency spectrum; able to penetrate the atmosphere and reach Earth’s surface. These wavelengths have attracted much attention in solar energy harvesting; thermography; and infrared imaging applications for the detection of electrical failures; faults; or thermal leakage hot spots and inspection of tapped live energized components. This paper presents a numerical analysis of a compact cubic cross-shaped four-layer metamaterial absorber (MA) structure by using a simple metal-dielectric-metal-dielectric configuration for wideband visible and infrared applications. The proposed MA achieved above 80% absorption in both visible and near-infrared regions of the spectrum from 350 to 1250 nm wavelength with an overall unit cell size of 0.57λ × 0.57λ × 0.59λ. The SiO_2_ based anti-reflection coating of sandwiched tungsten facilitates to achieve the wide high absorption bandwidth. The perceptible novelty of the proposed metamaterial is to achieve an average absorptivity of 95.3% for both visible and infrared wavelengths with a maximum absorptivity of 98% from 400 nm to 900 nm. Furthermore, the proposed structure provides polarization insensitivity with a higher oblique incidence angle tolerance up to 45°.

## 1. Introduction

Most of the energy from the sun arrives in the form of visible light and near-infrared electromagnetic radiation. The wavelengths measuring from 2 to 2.6 µm and 3 to 6 µm are also known as near- and mid-infrared Earth’s Atmospheric Transparency Window (ATW) [1]. Over the last decade, ATW research has drawn the attention of researchers after the concept of a perfect metamaterial absorber (PMA) in the gigahertz (GHz) frequency range was published [2,3]. Metamaterial absorbers (MA) in ATW offer various potential applications such as enhanced performance of solar radiation to energy conversion [4,5,6], radiation of heat into outer space [7], cooling of sky-facing objects [8], and direct electric power generation [9]. Moreover, atmospheric phenomena not related to radiation from space represent another subject of intensive research. Camouflage technology [10], invisible cloaks [11], antennas [12,13], super lenses [14], sensors [15,16,17,18,19], filters [20] and physical property detection [21] are some non-atmospheric applications. With the rapid improvement of simulation software and new measuring tools, real breakthroughs are being discovered every moment by researchers.

Metamaterial absorbers have some unique electromagnetic properties such as negative permeability and permittivity. In 1967, V. Veselago’s theoretical explanation [22] followed by Smith’s et al. illustration [23] showed that the properties of a metamaterial depend on the physical structure of the unit cell rather than the chemical properties of the metamaterial. By changing the shape or structure of a unit cell, absorption characteristics can be defined, enabling a variety of absorption-related applications [24,25]. Maintaining high absorption is usually achieved by introducing periodic unit cells [26]. The MA is also known as a perfect metamaterial absorber (PMA) when it completely absorbs a specific frequency [27].

Among the recent studies, in [28], a net-shaped graphene sheet MA for terahertz application is presented, achieving an absorption bandwidth from 1.27 THz to 2.59 THz. In [18], Chunlian Cen et al. presented a triple-band MA with three narrow absorption bands at 3.56 THz, 10.38 THz, and 12.96 THz with 99.57%, 99.98%, and 99.76% absorption, respectively. The article also discusses other MAs for solar-energy conversion, artificial colour, thermal emotion, and various optoelectronic applications. A broadband MA was discussed in [18], covering wavelengths larger than 1300 nm with more than 90% absorption. Periodic GaAs (gallium arsenide) was used on a tungsten film to construct the MA. On the other hand, a six-band terahertz MA was designed on an InSb substrate with a cross-cave metallic resonator that absorbs from 0.4 THz to 2.2 THz [21]. The peak absorption frequency of this MA can be altered by adjusting the temperature. As a result, it is useful for temperature sensing. A wideband metamaterial absorber for thermal-energy harvesting is shown in [25]. Within the bandwidths of 142–159 THz, 183–200 THz, and 233–245 THz, it bears an absorption of more than 80%. Although this particular MA has applications in thermal emission, photodetection, sensing, and solar energy harvesting, its bandwidth is quite restricted. Regarding the near-infrared region, an MA was presented in [29], in which a gold resonator is placed on a SiO_2_ substrate. However, this approach is not cost-effective as it requires a gold resonator. A broadband plasmonic light absorber based on a tungsten meander ring-resonator was designed and analyzed numerically in [30]. A multipole titanium layer was used in [26] to extend the absorption bandwidth, but this approach renders the fabrication process more complex. Researchers in [31] employ FR-4 as dielectric material and copper (Cu) in the bottom layer. However, both FR-4 and Cu are not transparent, which represents a disadvantage in the optical spectrum. Meanwhile, [32] utilize transparent materials such as Tu and SiO_2_, but their model did not achieve angular stability. A polarization and angle insensitive ultra-broadband MA for the infrared spectrum was presented in [33], although it contains a complex multilayer structure with a very thin chromium dielectric. As a result, the manufacture and subsequent fabrication of the MA is a challenging process. Furthermore, this MA is suitable for 800 nm to 4000 nm which covers the infrared spectrum but not the visible optical spectrum and is also polarization-insensitive up to 60°. An anisotropic plasmonic metasurface for the mid-infrared band was presented in [34]. This metasurface is highly sensitive to the oblique incidence angle and works as an absorber between 15° and 25° angle of oblique incidence, which is not ideal for broadband applications. It also employs gold, which increases the fabrication cost. Moreover, there are other works that achieve polarization insensitivity by using a water bubble [35], stair-like three-dimensional structured resonator [36] and tunable reconfigurable metasurface [37].

In [38], an ultrathin plasmonic absorber is presented. It uses gold (Au) as a resonating metal element making fabrication costly. Furthermore, these absorbers obtained only 71% in the visible spectrum from 400 to 700 nm with 45° incident angle stability. In [39], researchers employ a complex metal structure (gear-shape) of Fe. This structure can make the MA physically fragile. Moreover, Fe layer without any protective coating can cause oxidation. A silicon-based nanostructured polarization-insensitive broadband plasmonic absorber was studied in the article [40], which tolerates wide-incident angles for both TM and TE modes between 514 THz and 638 THz with 99.90% peak absorption. As common infrared absorbers, most earlier investigations have concentrated on multi-layered structures with alternating layers of metal and dielectric plates. This is performed in order to increase the absorption bandwidths. For example, to obtain absorption from 3000 to 5500 nm, [41] proposed an MA with 20 metal-dielectric layers. Despite the MA’s extensive infrared light absorption, the construction approach relies on expensive multipole nanofabrication processes. Furthermore, most investigations reveal that the polarization angle and incidence angle vary during practical use. This renders optical and infrared MA design more challenging to develop.

In addition, a simple MDM (metal-dielectric-meta) structured MA was described for optical and infrared regions of the spectrum. The uniqueness of the proposed structure is in its use of a small-size MA unit cell for both visible and near-infrared wavelength regions of the spectrum. The obtained absorber was further enhanced by introducing AR protective coating. A top layer of SiO_2_ acts as an anti-reflection protective coating that enhances the absorption performance and protects the Tu metal layer from oxidation. The average absorption over the optical and first infrared spectrum includes the range of 380–1250 nm reaching 95.3%. Moreover, the absorption of the developed MA is polarization-independent with a significant incident angle stable up to 45°. Further, a brief study of the electric field, magnetic field, and current density have been presented. The MA shows numerous excellent characteristics, including low cost, simple fabrication, simultaneous ultra-broad, and perfect absorption performances.

## 2. Unit Cell Design

**Figure 1:**
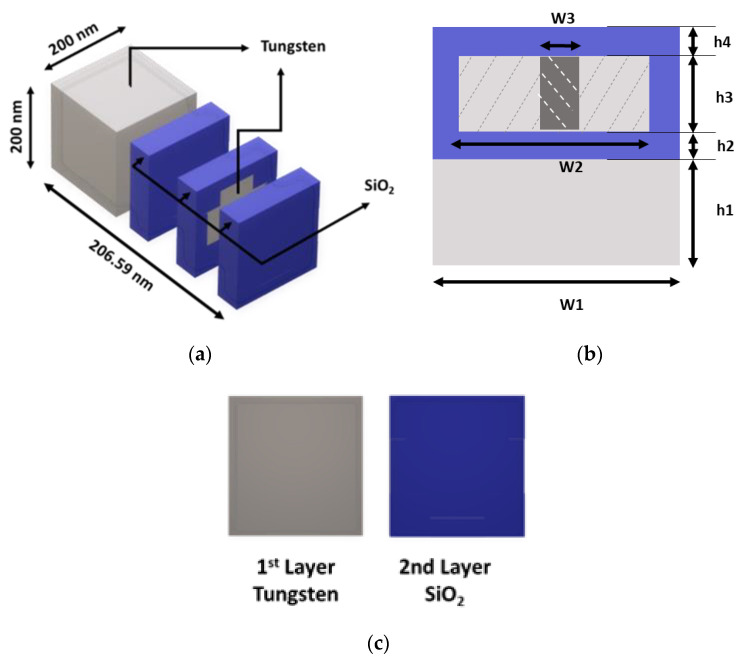
The design of the unit cell (**a**) perspective view (**b**) cross-section (**c**) internal structure and layer constituents.

**Figure 2:**
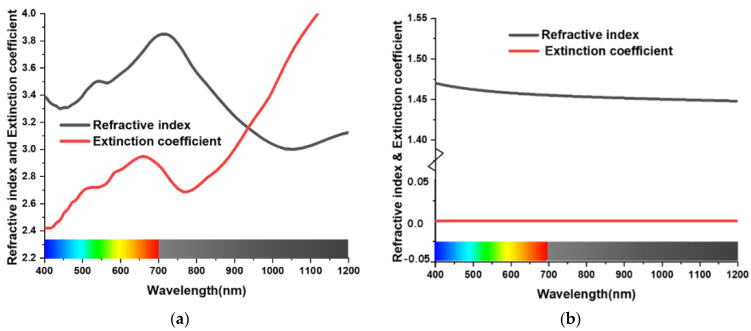
The refractive index and excitation coefficient of (**a**) tungsten and (**b**) SiO_2_ in the optical and infrared range.

Figure 1 depicts the unit cell design. The ultimate design of this cell contains four layers. The top layer is a SiO_2_ anti-reflection (AR) coating, and the bottom metal layer is tungsten (Tu), which acts as a reflector. There are two layers of SiO_2_ in between the AR and the reflective layer. One is the intermediate layer between the resonator and the ground, and the other is wrapped around the tungsten resonator. The final layer thicknesses are h1 = 105 nm, h2 = 12.5, h3 = 68, h4 = 21, respectively, resulting in a total thickness of 206.5 nm. The width of the proposed unit cell is W1 = 200 nm. Table 1 contains detailed structural information of MA. Simulation of the proposed MA unit cell was performed with CST Studio Suite [42]. Periodic boundary conditions have been applied for sidewall, where two floquet ports were applied at the top and bottom of the structure. Tungsten is substantially more dependable for a broad frequency band in the optical range and infrared range application than other commonly used metals in MA. At the same time, the use of tungsten can greatly reduce the production cost compared to the previous studies. For a typical sample of tungsten, the refractive index and excitation coefficient at 632.8 nm or mid optical window band are 3.63739 and 2.916877, respectively. On the other side of SiO_2_, the refractive index and excitation coefficient at 632.8 nm are 1.45704 and 0, respectively. Figure 2 shows the refractive index and excitation coefficient of tungsten and SiO_2_ in the optical and infrared range.

Later in this paper, different metals and media are compared. As the bottom layer comprises tungsten, the transmission of light is prevented. Therefore, the formula for absorption is represented by Equation (1) [29].

(1) A=1−R

where *A* is absorption and *R* is reflectance. In Figure 2, the optical parameters of Tungsten (Tu), and SiO_2_ are illustrated, obtained from [43,44,45]. For the vertical incident of electromagnetic wave, TM mode was used as the proceeding EM wave across the negative *z*-axis, and polarization of the proceeding light was aimed across the *x*-axis.

## 3. Result Analysis

**Figure 3:**
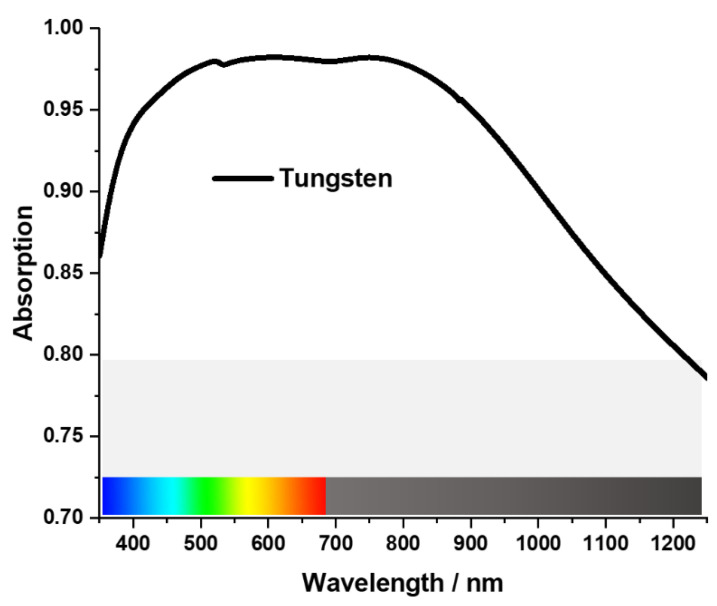
The Absorption of the MA.

The absorption of the proposed MA structure is illustrated in Figure 3. Over 90% absorption (nearly perfect absorption) was achieved by the MA in its targeted wavelengths. It achieved an average of 95.3% absorption throughout the wavelengths from 350 to 1250 nm. According to the simulated result, the proposed structure has a peak absorption of 98.2% at 602 nm wavelength.

**Figure 4:**
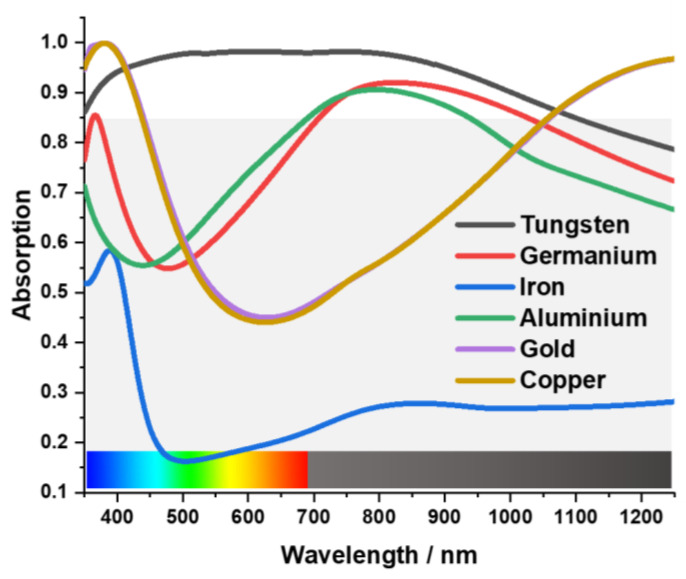
Comparison of different resonating components.

**Figure 5:**
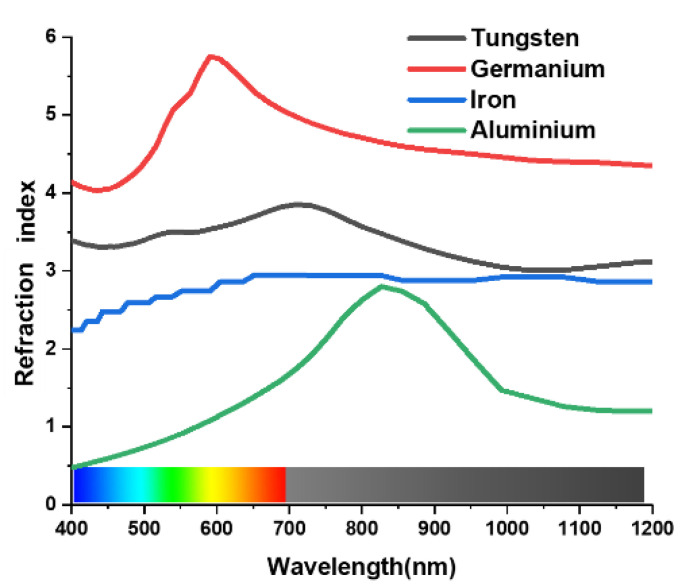
Refraction index of Tu, Ge, Al, and iron.

Considering the practical applications, wide bandwidth and high absorption rate are useful indicators when designing an absorber. An absorber must be insensitive to the large incident angle and independent of polarization. The proposed design was simulated and then improved gradually. The materials of the resonating metal surface bear a key influence on the absorption characteristic of the MA, which can be tested by using different materials alternatively. In Figure 4, a comparative study of different metals has been better performed to understand the absorption characteristic of the metamaterial unit cell. Tungsten represents an attractive material for PMA design in the optical spectrum, whereas germanium (Ge), aluminium (Al), and iron show lower absorption. The difference in refraction index of these materials is the reason behind this variation. Gold (Au) shows almost perfect absorption at 400 nm, but after 450 nm, the absorption decreases rapidly. Copper (Cu) demonstrates almost the same absorption characteristics. Figure 4 shows the comparison of different resonating components. For visible and infrared spectrum MA, the absorption and extensiveness of the bandwidth are only slightly affected by the refractive index of the metal [46]. Figure 5 shows the refraction index of Tu, Ge, Al, and iron.

**Figure 6:**
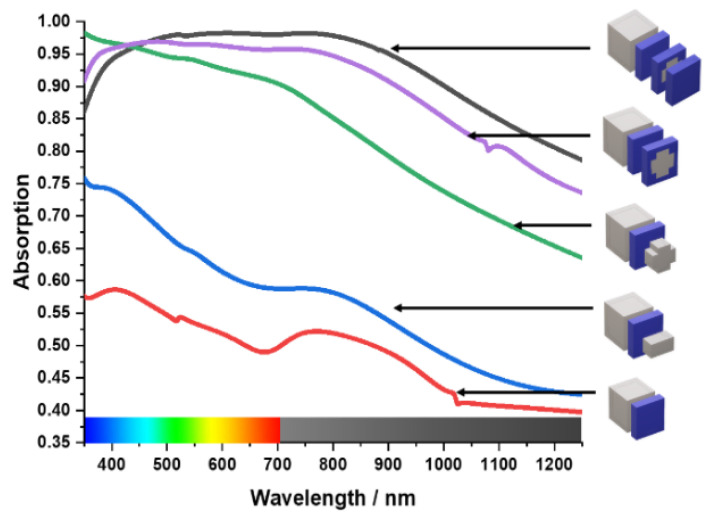
Design Evaluation of the proposed MA.

As with the materials, the MA’s absorption is related to the number of layers [47,48,49]. The proposed design is a four-layer MA. The absorption by different layers is shown in Figure 6. It can be seen that the increased number of layers also directly influence absorption. However, the addition of more layers renders the design process complex. As a result, there is a trade-off between layer count and absorption. In Figure 6, in comparison to the other structures, the final structure can provide a broader absorption bandwidth from 350 nm to 1250 nm. More importantly, the final MA structure covers the optical and near-infrared spectrum with polarization-independent and angle insensitive behaviour. Figure 6 shows the evaluation of the structure. The structure initially had only two layers. A bottom tungsten layer with a thickness of 105 nm and a top silicon dioxide layer with a thickness of 12.5 nm. This initial structure has an average absorption of 50% from 380 nm to 900 nm wavelength. Then, a tungsten bar with a length of 157.20 nm and width of 31.5 nm was introduced on top of the SiO_2_ layer. This new structure gave a 65% average absorption. Following this, another tungsten bar with the same length and width was placed on top of the SiO_2_ layer. However, this bar is 90^°^ clockwise rotated, forming a cross-like shape. The structure now starts to show better absorption compared to the previous structures. It has almost 97% absorption at 380 nm wavelength and an average of 80% absorption for the rest wavelength. However, as this layer is a metal surface, it reflects some of the incident light. As a result, this structure cannot absorb as expected. To solve this problem, a layer of SiO_2_ has been used to prevent reflection from the body of the metal cross. This gives a peak absorption of 95% from 400 nm to 800 nm. A layer of SiO_2_ has been introduced in the final structure to prevent reflection from the top. As a result, 97% absorption from 400 nm to 900 nm was finally achieved. The effect of SiO_2_ as an anti-reflection surface will be explained later on in this article.

**Figure 7:**
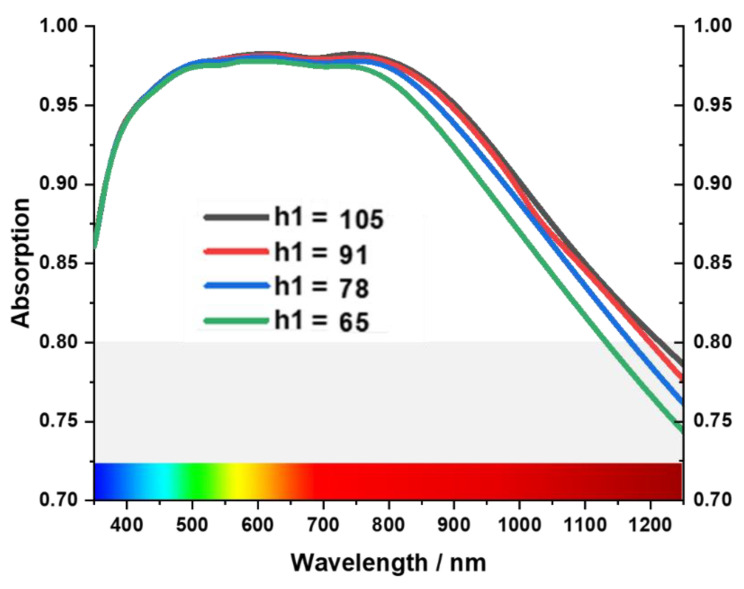
Effect of the thickness of the bottom tungsten layer.

**Figure 8:**
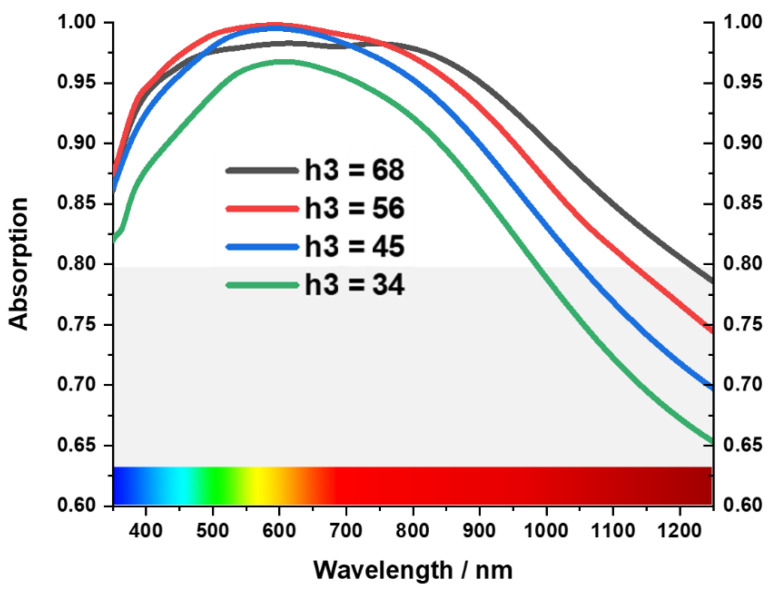
Effect of the thickness of resonating tungsten layer.

Tungsten acts as a metal blocking layer at the bottom of the MA, preventing waves from propagating through it [50]. The blocking capability of EM waves can be understood by skin depth $\delta =\sqrt{\rho /\pi f\mu }=\sqrt{\rho \lambda /\pi \mu }$, where wavelength is proportional to skin depth. So, a higher skin depth is required to block the larger wavelength [17,51]. Figure 7 shows the investigation of absorption property for different back layer thicknesses from h1 = 105 to 65, where the absorption is reduced in the near-infrared region with the decrease of the thickness h1. This phenomenon occurs due to the increase in EM transmission. On the other hand, the cross shape is the key resonant element of the MA, and h3 is the thickness of the tungsten cross resonator. As a result, the absorption is influenced by its thickness Figure 8 shows as the thickness (h3) decreases from 68 nm to 34 nm, absorption bandwidth shrinks from 900 nm to 650 nm (from 350 nm–1250 nm to 350 nm–950 nm) in a periodic manner. In terms of peak absorption, it remains almost the same up to h3 = 45 nm. However, when h3 is near 34 nm, peak absorption decreases to 95% from 98%.

The cross shape is a key resonant element of the MA, and h3 is the thickness of the tungsten cross resonator. As a result, the absorption is influenced by its thickness.

**Figure 9:**
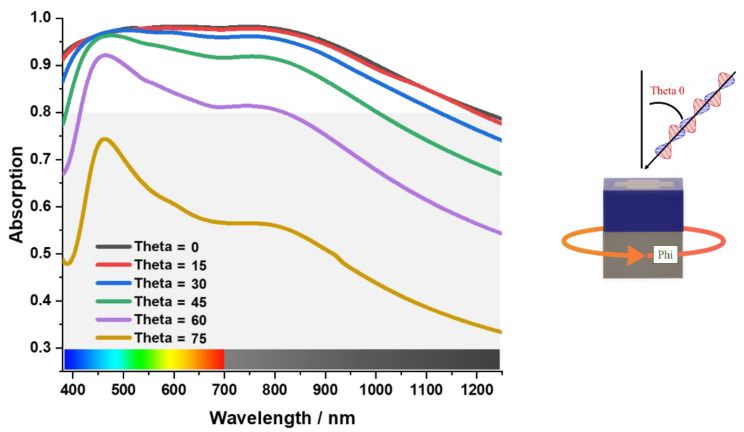
Angular-stability characteristics of the absorber.

**Figure 10:**
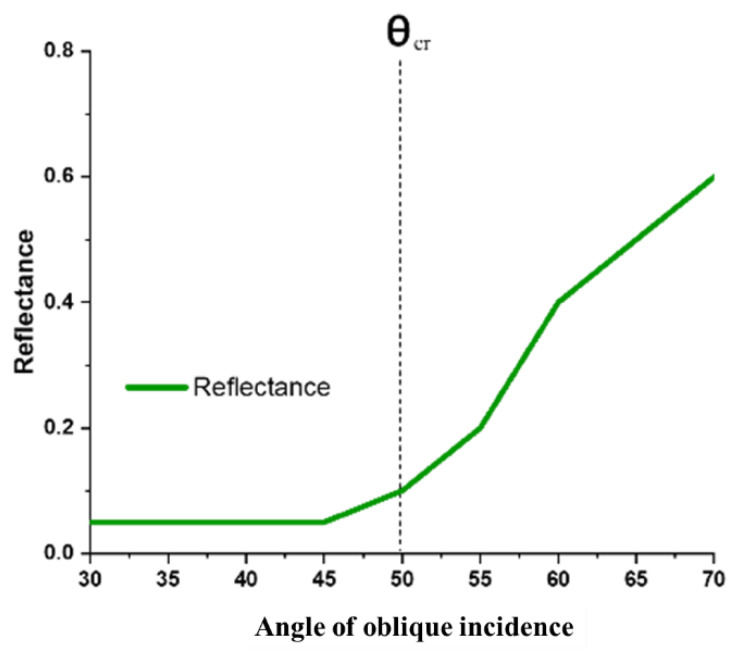
Critical angle of reflection of SiO_2_ [36].

Figure 9 shows the angular stability characteristics of the absorber. Here, θ (theta) is angle of oblique incidence. The simulated result shows that the absorber is stable up to “θ = 45°”. Beyond this, the MA starts to lose its angular stability, and becomes unstable after “θ = 75°”. The reason behind this is the SiO_2_ coating. In Figure 10, it can be seen that the critical angle of reflection of SiO_2_ is from “55°” in the infrared optical spectrum [36]. As a result, the incident light starts to reflect in the incident medium causing the MA to lose its absorption capacity. As a result, the MA becomes angular-unstable after “θ = 55°”. This represents a research opportunity for future studies.

**Figure 11:**
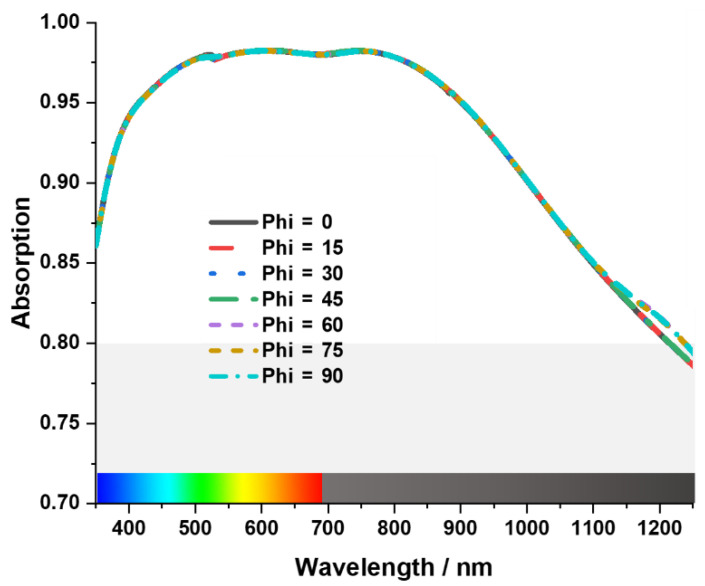
Illustration of the polarization independency of the MA.

Figure 11 illustrates the polarization independency of the proposed MA. It can be seen that the MA is polarization independent for the range “phi = 0°” to “phi = 90°”. There is a little change in absorption curve from “phi = 75°” to “phi = 90°”. However, it does not affect the overall absorption bandwidth of the MA.

**Figure 12:**
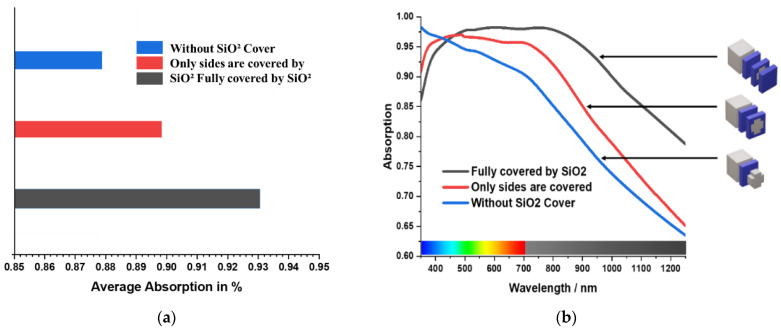
Illustration of the average absorption of three-component setups (**a**) average absorption (**b**) total absorption.

To demonstrate the anti-reflection effect of SiO_2_, the absorption spectrum of the MA was examined in three different component setups. Figure 12a illustrates the average absorption of three-component setups. It can be seen that the structure without any anti-reflection layer has the lowest average absorption of 88%. At the same time, the structure with a partial anti-reflection layer bears a comparatively better average absorption of 90%. However, the greatest average absorption of 93% was achieved by the structure with a complete SiO_2_ anti-reflection layer. Figure 12b illustrates the overall absorption of three-component setups with their corresponding structures.

**Figure 13:**
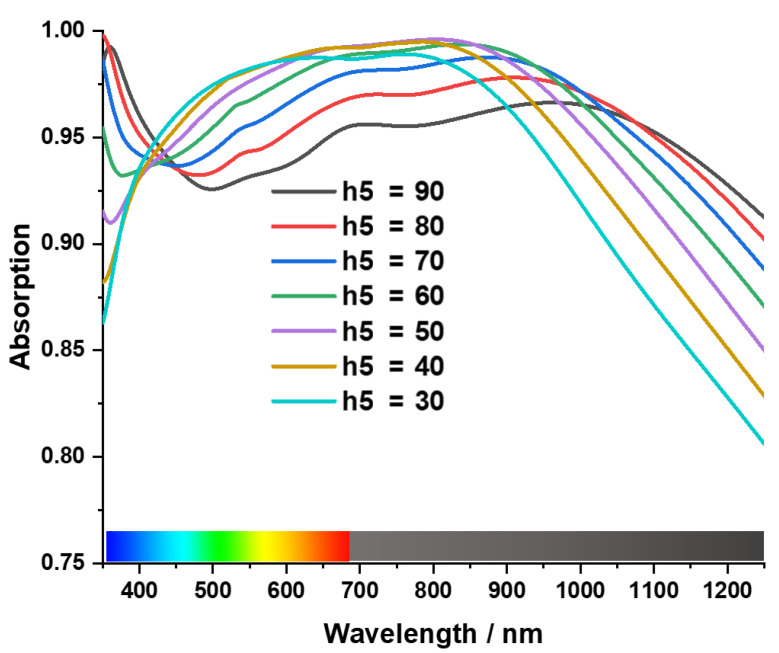
Comparison of SiO_2_ at different thickness.

A comparison of SiO_2_ at various thicknesses is shown in Figure 13. It is clear that the thickness of the SiO_2_ has an impact on the MA’s absorption capacity. The absorption bandwidth increases with increases in SiO_2_ thickness, while average peak absorption falls. If the thickness decreases, the opposite occurs. A thin SiO_2_ layer was employed in the final design of this study to keep the unit cell size to a minimum.

**Figure 14:**
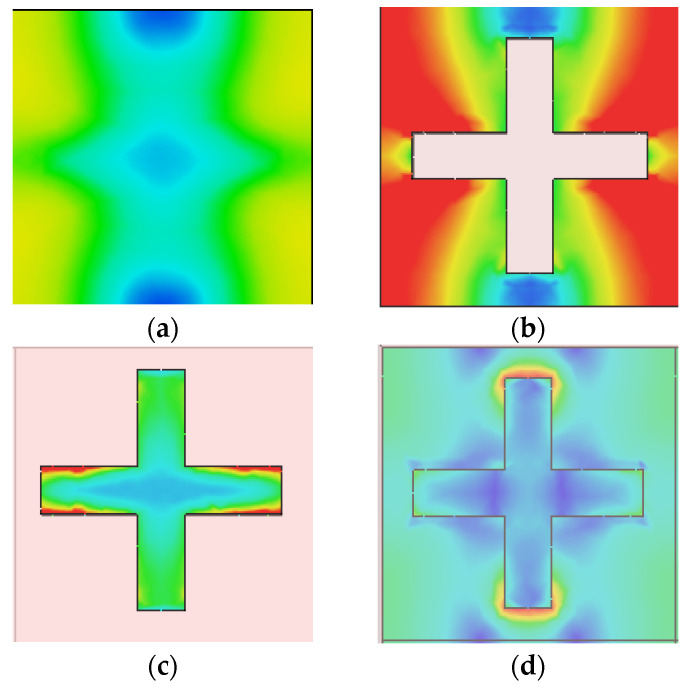

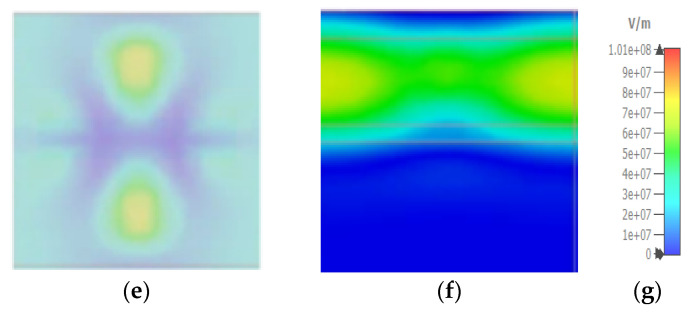
Electric field distribution at phase 560 (**a**) top SiO_2_ layer (**b**) SiO_2_ cover (**c**) Tungsten resonator (**d**) SiO_2_ dielectric layer (**e**) Bottom tungsten layer (**f**) Side view of the MA unit cell (**g**) unit in V/m.

**Figure 15:**
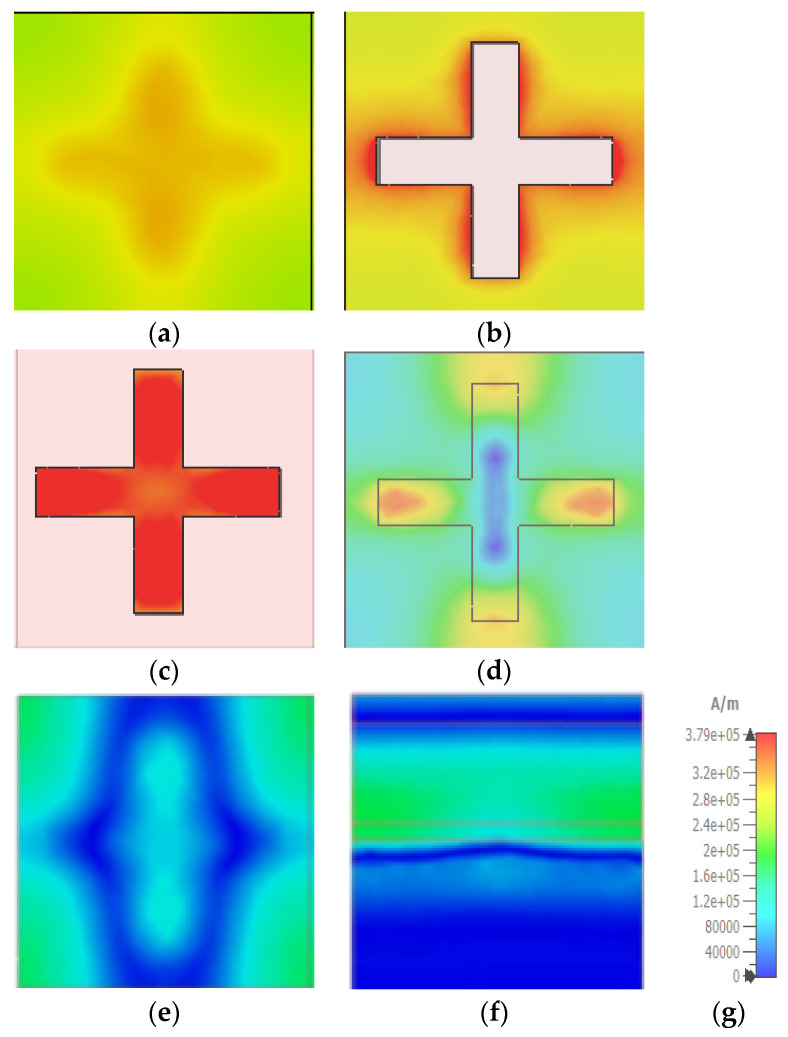
Magnetic field distribution at phase 560 (**a**) top SiO_2_ layer (**b**) SiO_2_ cover (**c**) Tungsten resonator (**d**) SiO_2_ dielectric layer (**e**) Bottom tungsten layer (**f**) Side view of the MA unit cell (**g**) Unit in A/m.

The electric and magnetic fields (EF and HF) are discussed below to understand the proposed MA’s absorption mechanism better. Figure 14a–f and Figure 15a–f show that the EM field is intensified at some regions of the MA peak absorption point wavelength. A dipolar field density has formed between the metal resonator and the metal bottom layer. It can be seen that EF and HF density formed perpendicularly in different structure positions for the same wavelength. HF is highly induced in the insulating layer due to resonance of the surface plasmons. The outer corners of the Tu-cross experience greater excitation than the middle. The cavity gap between the surrounding Tu crosses of other unit cells is one of the reasons behind this.

**Figure 16:**
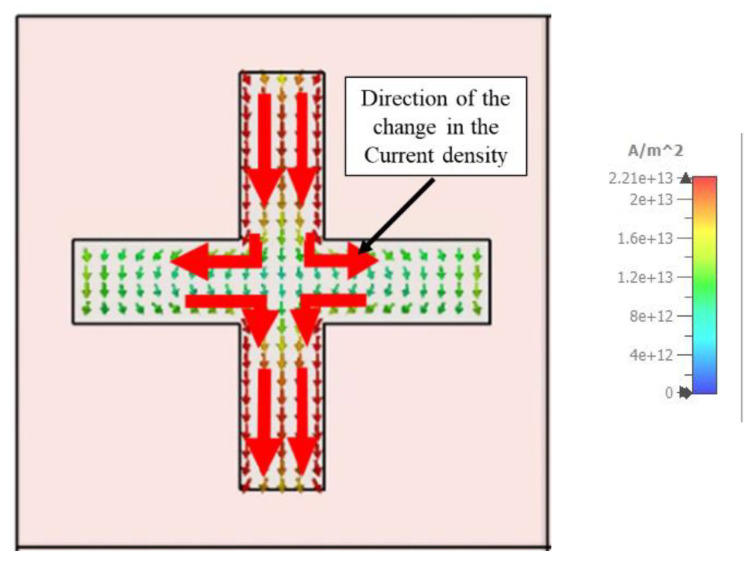
The current mode of the metal layer at 450 nm wavelength.

**Figure 17:**
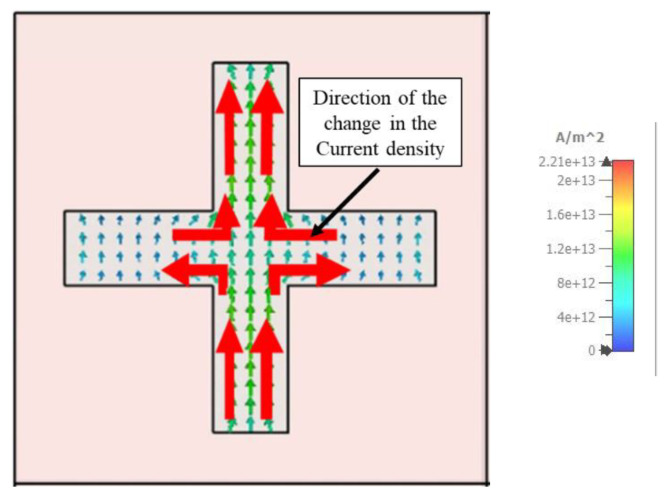
The current mode of the metal layer at 1150 nm wavelength.

As illustrated in Figure 16 and Figure 17, the resonance mechanism is explored by comparing the surface current of the metal layer at 450 nm and 1150 nm wavelengths, respectively. At 450 nm, there is a large polarization rotation impact, but at 1150 nm, there is a negligible polarization rotation effect. The surface current is estimated using a linearly polarized incidence oriented on the *x*-axis, with red arrows indicating the current flow direction.

Table 2 shows the comparison between previous studies and the presented MA structure. It can be seen that the presented MA bears the most considerable absorption bandwidth, and a relatively good average absorption spectrum while only using two cost-efficient constituents with a four-layer structure. This MA covers the optical range entirely and the near-infrared spectrum. Most remarkably, it bears the smallest size among other MA structures.

## 4. Conclusions

An optical and infrared window MA consisting of metal-dielectric with metal anti-reflection coating was theoretically obtained and studied. The proposed MA revealed an average absorption of 95.3% from 350 nm to 1250 nm wavelength, corresponding to the optical range and the near-infrared spectrum. Furthermore, the MA achieved complete polarization insensitivity and an angular stability up to 45°. The physical properties of perfect broadband absorption were thoroughly investigated. The proposed MA with a simple structure shows a larger absorption band of 900 nm, peak absorption of 98%, and the smallest size of 0.57λ × 0.57λ × 0.59λ. These properties render the proposed MA suitable for optical and infrared window applications. Moreover, this study provides guidelines for future ATW research.

## Figures and Tables

**Table 1:** Structural parameters of the MA unit cell.

Symble	Parameter Value (nm)	Symble	Parameter Value (nm)
W1	200	h2	12.5
W2	157.20	h3	68
W3	31.5	h4	21
h1	105		

**Table 2:** Comparison table.

Ref.	Operating Band (THz)	WL- nm	Avg. Absorption	Polarization Independency Angular Stability	Materials	No. of Layer	Dimension (Length × Width × Height)
[29]	300–516	580–1000	95.2%	Independent, θ = 0°	Ge_2_, Sd_2_, Te_5_	2	0.4λ × 0.4λ × 0.21λ
[30]	0.1–3	2.9 × 10^6^–9.993 × 10^4^	87.09%	Independent, θ = 0°	Cu, Si	4	5λ × 5λ × 0.15λ
[27]	21–37	8000–14,000	95%	Independent	Ti, Si	5	0.3λ × 0.3 λ × 0.1λ
[40]	24.58–28.82	10,000–12,000	88%	Independent, θ = ≤50°	In_2_SnO_5_, ZnS	4	0.8 λ × 0.8 λ × 0.055 λ
[31]	80–160	1800–3700	98%	Independent, θ = ≤45°	GaSa, FR-4, Cu	4	5λ × 5λ × 0.4λ
[32]	428–1070	280–700	90%	Independent, θ = ≤30°	Tungsten, SiO_2_	3	2λ × 2λ × 0.4λ
[30]	100–666	450–3000	98%	Independent,	Fe, Si, Au	3	0.6λ × 0.6λ × 2λ
[1]	220–360	2000–6000	89%	Independent, θ = ≤35°	Cu, GaAs	4	0.77λ × 0.77λ × 0.11λ
[52]	428–750	300–700	92.2%	Independent θ = ≤70°	Tungsten, SiO_2_	3	3.2 λ × 3.2λ × 0.77λ
[38]	430–750	400–700	71%	Independent θ = ≤45°	Ag, SiO_2_	3	0.75 λ × 0.75 λ × 0.65 λ
Proposed MA	240–856	350–1250	95.3%	Independent θ = ≤45°	Tungsten, SiO_2_	4	0.57λ × 0.57λ × 0.59λ

## Data Availability

The data presented in this study are presented in this article.
